# Supplementary material for: Epidemiology and severity risk factors of dengue virus infection during the 2023-2024 outbreak in Colombia
Source: PLoS Negl Trop Dis. 2025 Dec 5;19(12):e0013115. doi: 10.1371/journal.pntd.0013115 (PMC12694831; doi:10.1371/journal.pntd.0013115)
Supplement: S1 Table — (DOCX) [file pntd.0013115.s001.docx]

**Supplementary Table 1. Sociodemographic and clinical characteristics of patients with Non severe and severe dengue virus infection according to WHO classification**

| **Variable** | **Non-Severe (WHO)**  **N=578** | **Severe (WHO)**  **N=22** | **Total**  **N=600** | **OR [95CI]** | **p- value** |
| --- | --- | --- | --- | --- | --- |
| Age of patient, median years (IQR) | 13.0 (8.0 to 19.0) | 10.5 (7.5 to 21.2) | 13.0 (8.0 to 19.0) | 1 [0.98, 1.03] | 0.715 |
| Sex |  |  |  |  |  |
| Female | 262 (45.3) | 10 (45.5) | 272 (45.3) |  |  |
| Male | 316 (54.7) | 12 (54.5) | 328 (54.7) | 0.99 [0.42, 2.39] | 0.991 |
| Race |  |  |  |  |  |
| White | 88 (15.2) | 4 (18.2) | 92 (15.3) | - |  |
| Black | 46 (8.0) | 3 (13.6) | 49 (8.2) | 1.43 [0.27, 6.77] | 0.646 |
| Mix race | 444 (76.8) | 15 (68.2) | 459 (76.5) | 0.74 [0.26, 2.65] | 0.606 |
| Level of education |  |  |  |  |  |
| Less than basic | 262 (45.3) | 11 (50.0) | 273 (45.5) | - |  |
| Basic/intermediate | 247 (42.7) | 8 (36.4) | 255 (42.5) | 0.77 [0.29, 1.94] | 0.583 |
| Advanced | 69 (11.9) | 3 (13.6) | 72 (12.0) | 1.04 [0.23, 3.42] | 0.958 |
| Place of origin |  |  |  |  |  |
| Urban | 528 (91.3) | 21 (95.5) | 549 (91.5) | - |  |
| Rural | 50 (8.7) | 1 (4.5) | 51 (8.5) | 0.5 [0.03, 2.48] | 0.506 |
| City of origin |  |  |  |  |  |
| Cali | 355 (61.4) | 7 (31.8) | 362 (60.3) | - |  |
| Other | 223 (38.6) | 15 (68.2) | 238 (39.7) | 3.41 [1.42, 9.05] | 0.008 |
| Occupation |  |  |  |  |  |
| House wive | 41 (7.1) | 2 (9.1) | 43 (7.2) | - |  |
| Employee | 65 (11.2) | 2 (9.1) | 67 (11.2) | 0.63 [0.07, 5.42] | 0.651 |
| Freelance work | 14 (2.4) | 1 (4.5) | 15 (2.5) | 1.46 [0.07, 16.5] | 0.763 |
| Unemployed/none | 86 (14.9) | 5 (22.7) | 91 (15.2) | 1.19 [0.25, 8.57] | 0.838 |
| Student | 372 (64.4) | 12 (54.5) | 384 (64.0) | 0.66 [0.17, 4.34] | 0.597 |
| Body Mass Index |  |  |  |  |  |
| Underweight | 38 (6.6) | 2 (9.1) | 40 (6.7) | 1.36 [0.20, 5.52] | 0.703 |
| Normal | 232 (40.1) | 9 (40.9) | 241 (40.2) | - |  |
| Overweight | 123 (21.3) | 5 (22.7) | 128 (21.3) | 1.05 [0.32, 3.10] | 0.934 |
| Obese | 185 (32.0) | 6 (27.3) | 191 (31.8) | 0.84 [0.28, 2.36] | 0.738 |
| Any comorbidity | 88 (15.2) | 6 (27.3) | 94 (15.7) | 2.09 [0.73, 5.23] | 0.135 |
| Time fever to consult (days) | 3.0 (1.0 to 4.0) | 3.0 (1.0 to 4.0) | 3.0 (1.0 to 4.0) | 1.11 [0.87, 1.39] | 0.366 |
| ***Clinical symptoms*** |  |  |  |  |  |
| Headache | 510 (88.2) | 20 (90.9) | 530 (88.3) | 1.33 [0.38, 8.47] | 0.702 |
| Retro-ocular pain | 396 (68.5) | 15 (68.2) | 411 (68.5) | 0.98 [0.41, 2.62] | 0.974 |
| Mialgias | 506 (87.5) | 21 (95.5) | 527 (87.8) | 2.99 [0.61, 54.0] | 0.288 |
| Arthralgias | 496 (85.8) | 22 (100.0) | 518 (86.3) | - | - |
| Rash | 315 (54.5) | 10 (45.5) | 325 (54.2) | 0.7 [0.29, 1.64] | 0.406 |
| Vomiting | 391 (67.6) | 16 (72.7) | 407 (67.8) | 1.28 [0.52, 3.60] | 0.617 |
| Abdominal pain | 461 (79.8) | 17 (77.3) | 478 (79.7) | 0.86 [0.33, 2.67] | 0.776 |
| Diarrhea | 237 (41.0) | 12 (54.5) | 249 (41.5) | 1.73 [0.73, 4.15] | 0.211 |
| Bleeding | 201 (34.8) | 9 (40.9) | 210 (35.0) | 1.3 [0.53, 3.06] | 0.555 |
| Petechiaes | 308 (53.3) | 9 (40.9) | 317 (52.8) | 0.61 [0.25, 1.43] | 0.258 |
| Edema | 124 (21.5) | 14 (63.6) | 138 (23.0) | 6.41 [2.68, 16.4] | <0.001 |
| ***Laboratory characteristics*** |  |  |  |  |  |
| Leukocytes | 4.6 (3.0 to 7.0) | 6.7 (4.6 to 12.7) | 4.6 (3.1 to 7.0) | 6.41 [2.68, 16.4] | <0.001 |
| Neutrophils | 1.8 (1.1 to 3.2) | 2.9 (1.7 to 6.8) | 1.8 (1.1 to 3.1) | 1.17 [1.09, 1.25] | <0.001 |
| Lymphocytes | 1.9 (1.0 to 3.2) | 2.4 (1.6 to 3.8) | 1.9 (1.0 to 3.2) | 1.19 [1.09, 1.29] | <0.001 |
| Platelets | 72.0 (39.0 to 150.0) | 36.0 (25.5 to 89.5) | 71.5 (38.0 to 147.5) | 1.18 [0.97, 1.41] | 0.078 |
| ALT | 58.0 (30.0 to 118.2) | 150.0 (37.5 to 569.7) | 58.0 831.0 to 119.7) | 0.99 [0.98, 1.00] | 0.069 |
| AST (columna AY) | 112.0 (61.0 to 201.0) | 233.5 (108.7 to 878.2) | 112.0 (61.0 to 214.0) | 1 [1.00, 1.00] | 0.001 |
| IgG |  |  |  |  |  |
| Negative | 205 (38.9) | 9 (45.0) | 214 (39.1) | - |  |
| Positive | 322 (61.1) | 11 (55.0) | 333 (60.9) | 0.78 [0.32, 1.96] | 0.584 |
| Dengue serotype |  |  |  |  |  |
| Unknown | 248 (42.9) | 12 (54.5) | 260 (43.3) | 1.29 [0.51, 3.53] | 0.597 |
| DENV-1 | 52 (9.0) | 1 (4.5) | 53 (8.8) | 0.51 [0.03, 2.98] | 0.538 |
| DENV-2 | 187 (32.4) | 7 (31.8) | 194 (32.3) | - | - |
| DENV-3 | 89 (15.4) | 2 (9.1) | 91 (15.2) | 0.6 [0.09, 2.54] | 0.53 |
| DENV-4 | 2 (0.3) | 0 (0.0) | 2 (0.3) |  |  |
| Dengue lineage |  |  |  |  |  |
| 1V_D | 1 (0.2) | 0 (0.0) | 1 (0.2) |  |  |
| 1V_D.1 | 33 (5.7) | 0 (0.0) | 33 (5.5) |  |  |
| 1V_D.1.1 | 2 (0.3) | 0 (0.0) | 2 (0.3) |  |  |
| 1V_D.2 | 9 (1.6) | 1 (4.5) | 10 (1.7) |  |  |
| 1V_F | 1 (0.2) | 0 (0.0) | 1 (0.2) |  |  |
| 2II_F.1 | 1 (0.2) | 0 (0.0) | 1 (0.2) |  |  |
| 2II_F.1.1.2 | 108 (18.7) | 4 (18.2) | 112 (18.7) |  |  |
| 2II_F.1.1.5 | 2 (0.3) | 0 (0.0) | 2 (0.3) |  |  |
| 2III_D.2 | 61 (10.6) | 3 (13.6) | 64 (10.7) |  |  |
| 3III_B.3 | 1 (0.2) | 0 (0.0) | 1 (0.2) |  |  |
| 3III_B.3.2 | 1 (0.2) | 0 (0.0) | 1 (0.2) |  |  |
| 3III_C.1 | 85 (14.7) | 2 (9.1) | 87 (14.5) |  |  |
| 4II_B | 1 (0.2) | 0 (0.0) | 1 (0.2) |  |  |
| Unknown | 272 (47.1) | 12 (54.5) | 284 (47.3) |  |  |
| Dengue lineage |  |  |  |  |  |
| Other/unknown | 291 (50.3) | 13 (59.1) | 304 (50.7) | - |  |
| 1V_D.1 | 33 (5.7) | 0 (0.0) | 33 (5.5) | - |  |
| 2II_F.1.1.2 | 108 (18.7) | 4 (18.2) | 112 (18.7) | 0.83 [0.23, 2.40] | 0.748 |
| 2III_D.2 | 61 (10.6) | 3 (13.6) | 64 (10.7) | 1.10 [0.25, 3.54] | 0.883 |
| 3III_C.1 | 85 (14.7) | 2 (9.1) | 87 (14.5) | 0.53 [0.08, 1.96] | 0.405 |
