# Supplementary material for: Epidemiology and severity risk factors of dengue virus infection during the 2023-2024 outbreak in Colombia
Source: PLoS Negl Trop Dis. 2025 Dec 5;19(12):e0013115. doi: 10.1371/journal.pntd.0013115 (PMC12694831; doi:10.1371/journal.pntd.0013115)
Supplement: Attachment — Submitted filename: Response to Reviewers(PNTD-D-25-00700).docx [file pntd.0013115.s003.docx]

October 15, 2025

Doctors:

Viviane Sampaio Boaventura
Academic Editor
PLOS Neglected Tropical Diseases

David Safronetz
Section Editor
PLOS Neglected Tropical Diseases

Shaden Kamhawi
co-Editor-in-Chief
PLOS Neglected Tropical Diseases

Paul Brindley
co-Editor-in-Chief
PLOS Neglected Tropical Diseases

Re: Manuscript Revision PNTD-D-25-00700-Epidemiology and severity risk factors of dengue virus infection during the 2023-2024 outbreak in Colombia.

Dear all

We appreciate the opportunity to respond to the reviewer’s valid points. We believe we have responded to each below and are happy to provide further clarification where needed. I am submitting a revised version that incorporates the comments of the reviewer.

The specific comments and responses follow:

**Main**

Reviewer #1

1. Univariable selection (using variables with p < 0.05) is not a recommended approach for variable selection, as it often misses important variables in the model.
   (<https://pubmed.ncbi.nlm.nih.gov/8699212/> \ <https://pubmed.ncbi.nlm.nih.gov/27896874/> \ <https://onlinelibrary.wiley.com/doi/full/10.1002/bimj.201700067> ). Using background knowledge typically yields more stable and useful models. Alternatively, backwards elimination can be performed.

   The decision to reuse the data for two different types of models is also not appropriate, as the model with signs/symptoms will lack all the important demographic variables, such as age.

**Response:** Thank you for your suggestion, we have proceeded accordingly and modified the statistical analysis (lines 175-185). All variables measured in this study were selected based on prior knowledge. The revised multiple logistic model was proposed to identify risk factors associated with dengue with serious manifestations using a descriptive approach, as suggested by Heinze et al. (<https://onlinelibrary.wiley.com/doi/10.1002/bimj.201700067>). However, there is a restriction on the number of covariates allowed in the model, since the number of cases with serious manifestations of dengue in this study was 167. Therefore, if we implemented the rule of thumb of 10 events per variable (EPV), a maximum of 16 coefficients can be estimated in a single multiple model.

After careful review of your suggestion (and the other reviewers’), and the articles that were shared related to variable selection, the following modifications were made:

- A single multiple logistic model was created to simultaneously consider demographic characteristics, laboratory characteristics, and clinical symptoms. Furthermore, serotype was not recoded, and all serotypes (DENV-1, DENV-2, and DENV-3) were included in the final model.
- Since this is a descriptive model, we do not have a causal or predictive objective. Therefore, we decided to build a multiple model with those variables with a p-value <0.25 in the univariate model without performing any selection algorithm (e.g., backward or forward), as suggested by Heinze et al. This decision was made mainly because we have an EPV<10, and in this situation, variable selection is not recommended.

2. This strategy of univariable selection can also generate spurious associations; for example, the association between education level and serious dengue. Considering the age group (median 13, IQR 8-19) evaluated, education level will be highly collinear with age, so individuals with an “Advanced” education level (I’m considering that it is a college degree, as there is no description in the methods section) will be at least around 23 years old. Using the data provided in the submission, individuals with “Advanced” education have a median age of 30 years (20 to 40), while those with basic-intermediate education have a median age of 16 (13 to 20). Any discussion related to this should regard age difference as the main mechanism, contrary to the hygiene hypothesis, which is discussed.

**Response:** We agree and thank the reviewer for highlighting this issue. The discussion has been modified. Considering the lack of association in the final model between level of education and serious manifestations, the “hygiene hypothesis paragraph” has been deleted from the discussion. In addition, we have provided definitions for “level of education” as a footnote in Tables 1 and 2.

3. Considering that both education level and occupation are highly correlated with age (within the age group of the study participants), a better variable to adjust for would be the socioeconomic status (income) of the household.

**Response:** We acknowledge that direct measures of income or overall socioeconomic status (SES) may provide stronger predictive power for risk of severe dengue. However, during the pilot phase of cohort creation, we found income to be highly sensitive and subject to non‐response, and thus difficult to collect reliably. As a result, we used proxy indicators of SES—namely education level and occupational category. In our multivariate analysis, neither of these proxies showed a statistically significant association with serious manifestations of dengue after adjustment for covariates.

4. There is also a strong effect of city of residence, with areas outside Cali being prognostic of serious dengue. If more severe cases are referred to the hospital, this variable merely reflects the data generation mechanism, rather than any biological effect of living outside Cali.

**Response:** We thank the reviewer for pointing this out. We have now acknowledged this important limitation.

Reviewer #2

1. Which gene is used for dengue virus genotyping?

**Response:** Dengue virus genotyping and lineage designations were made using variable regions across the entire genome. As stated in the methods, we used the Genome Detective and Nextclade tools that are validated for this purpose using sequences that cover at least 5% of the genome. The exact part of the genome that is covered does not usually matter if there is divergence. If the regions covered cannot differentiate between genotypes or lineages, then the result will be "undetermined". The system is described in detail in the following reference: Hill V, Cleemput S, Pereira JS, Gifford RJ, Fonseca V, Tegally H, et al. A new lineage nomenclature to aid genomic surveillance of dengue virus. PLoS Biol. 2024;22(9):e3002834.

**Results**

Reviewer #1

1. Table 1 should also include a breakdown of comorbidities, even if they are not referred to as individual terms due to sample size constraints, enabling the reader to assess which types of comorbidities are being evaluated. Table 1 should clearly indicate the presence of missing data (for example, IgG only has data for 547 patients)

**Response:** All comorbidities were added to Table 1. In addition, the number of non-missing observations for each variable with missing values was reported.

2. It is also unclear what the interaction model is. (What are the reference levels in Table 3?) Considering the model: Serious Dengue ~ Current infection * previous infection, it should present only one p-value evaluating the joint effect (Wald-test / likelihood test) instead of the individual p-values of the interactions (<https://stats.oarc.ucla.edu/other/mult-pkg/faq/general/faqhow-are-the-likelihood-ratio-wald-and-lagrange-multiplier-score-tests-different-andor-similar/>). Moreover, the current p-values in Table 3 are also unclear, as there is significant overlap between the CIs for Dengue-2 despite p=0.05. Additionally, it is uncertain whether the interaction model incorporates all sociodemographic characteristics or only the current and previous infections.

**Response:** Thank you for your suggestion. Accordingly, and also considering the recommendations of reviewer #3 (to include all serotypes in the models), the following modifications were made:

The serotype variable was not recoded, and all serotypes (DENV-1, DENV-2, and DENV-3) were included in the final model. Only DENV-4 was excluded due to the low number of observations (n = 4).

We assessed the interaction between all serotypes and history of dengue infection (IgG) with respect to dengue severity, incorporating in the analysis age, sex, city of origin, education level, and occupation. Using a likelihood ratio test, we found that the interaction was not statistically significant and this has been reported in the result (lines 290-293) and discussion (lines 366-371) sections.

Reviewer #2

1. The OR merely reflects the strength of association between exposure and outcome but does not in itself establish a “high-risk factor.” ORs between 2–3 typically represent moderate risk and are common in clinical studies. Therefore, while risk factors such as residence outside Cali, DENV-2 infection, edema, bleeding, and thrombocytopenia appear statistically reliable, the interaction term between IgG status and DENV-2 infection remain inconclusive and requires further validation.

**Response:** Thank you for your comment. A similar issue was brought up by reviewers 1 and 3. Accordingly, the following modifications were made:

The serotype variable was not recoded, and all serotypes (DENV-1, DENV-2, and DENV-3) were included in the final model. Only DENV-4 was excluded due to the low number of observations (n = 4).

We assessed the interaction between serotype and history of dengue infection (IgG) with respect to dengue severity, incorporating in the analysis age, sex, city of origin, education level, and occupation. Using a likelihood ratio test, we found that the interaction was not statistically significant and this has been reported in the result (lines 290-293) and discussion (lines 366-371) sections.

2. The multivariable model relies on variables selected solely based on univariate P values, which increases the risk of overfitting. It would be more appropriate to use clinically justified variables, along with diagnostics for collinearity (e.g., VIF) and model fit indices (e.g., AIC/BIC). As such, I consider the conclusions of this study somewhat overstated.

**Response:** We thank the reviewer for this helpful suggestion. According to your concern, and reviewer’s 1, we have proceeded accordingly and modified the statistical analysis (lines 175-185): All variables measured in this study were selected based on prior knowledge. The revised multiple logistic model was proposed to identify risk factors associated with dengue with serious manifestations using a descriptive approach, as suggested by Heinze et al. (<https://onlinelibrary.wiley.com/doi/10.1002/bimj.201700067>). However, there is a restriction on the number of covariates allowed in the model, since the number of cases with serious manifestations of dengue in this study was 167. Therefore, if we implemented the rule of thumb of 10 events per variable (EPV), a maximum of 16 coefficients can be estimated in a single multiple model.

After careful review of your suggestion (and the other reviewers’), and the articles that were shared related to variable selection, the following modifications were made:

1. A single multiple logistic model was created to simultaneously consider demographic characteristics, laboratory characteristics, and clinical symptoms. Furthermore, serotype was not recoded, and all serotypes (DENV-1, DENV-2, and DENV-3) were included in the final model.
2. Since this is a descriptive model, we do not have a causal or predictive objective. Therefore, we decided to build a multiple model with those variables with a p-value <0.25 in the univariate model without performing any selection algorithm (e.g., backward or forward), as suggested by Heinze et al. This decision was made mainly because we have an EPV<10, and in this situation, variable selection is not recommended.
3. Multicollinearity was assessed using the variance inflation factor, as stated in methods.

The study’s conclusions in the abstract and in the body of the manuscript have been modified to better reflect the findings of the new model.

Reviewer #3

1. Figure 1 - Where the data were extracted from for the period 2021-2024. The methodology and results were unclear.

**Response:** Thank you for pointing this out. We have tried to be clearer in the methods (Study design, Participants and Setting – lines 130-134 and analysis – lines 185-188) and results section (lines 190-192).

2. Figure 1 - How the 400% value was reached was unclear.

**Response:** Thank you for helping us to clarify this finding. The average number of VCD cases during the epidemic period (April 2023–2024) was compared to that of the 2021–March 2023 period. This has now been included in the analysis section.

3. Table 2a - I suggest including all serotypes in the regression analysis, not just DENV-2.

**Response:** We agree. In the current version, the serotype was not recoded, and all serotypes (DENV-1, DENV-2, and DENV-3) were included in the final model. Only DENV-4 was excluded due to the low number of observations (n = 4).

4. Lines 311-313. This refers to the group of IgG-positive patients. Is this correct? Make this clear in the text.

**Response:** Yes, it refers to IgG-positive patients. This has been clarified.

5. Table 3. Perform the analysis separately for the full cohort and the sub cohort of seropositive patients. Include a supplementary table with the sub cohort of seropositive patients.

**Response:** Thank you for your comment. After we implemented all the changes requested by the reviewers (i.e. removing recoding across serotypes and using a single model adjusted for sociodemographic and clinical covariates), the interaction between serotype and history of dengue infection was no longer significant. Accordingly, we did not perform stratified analyses by serostatus in the current version. We can provide those analyses if the reviewer or Editor prefers.

6. Table 1 - What statistical test was used to generate the OR?

**Response:** Univariate and multivariable logistic regression models. This is described in the methods section (analysis, lines 182-185).

7. Table 1 - Present the limitation of unidentified serotypes in each group. Discuss this limitation as well.

**Response:** Thank you for this suggestion, we agree. This has now been acknowledged in the results section, lines 261-262 and in the limitations section.

8. Table 2a - Include other serotypes in the analysis as well, not just DENV-2.

**Response:** Agree. The serotype was not recoded in the current version, and all serotypes (DENV-1, DENV-2, and DENV-3) were included in the final model. Only DENV-4 was excluded due to the low number of observations (n = 4).

9. Table 1 - There is a difference in the city between the severe and non-severe groups. Perform the analysis with the variables separately for the city of Cali and other cities, as this may be a confounding factor.

**Response:** Thank you for this comment. Reviewer #1 also raised the issue of city of origin; accordingly, we have added a paragraph to the Limitations section.

As suggested, we conducted stratified analyses by city (Cali vs. other cities). As expected with smaller subgroup sample sizes, confidence intervals widened, but the odds ratios were directionally consistent and of similar magnitude in one or both strata as compared to the original analysis (see below).

Because the study was not designed to estimate causal effects, we did not perform confounder adjustment and have not included these tables in the current manuscript to maintain focus. We will be happy to provide them (in the main text or as supplementary material) if the reviewer or editor prefers.

**Sociodemographic and clinical characteristics of patients with serious and non-serious manifestations of dengue in the city of Cali.**

| **Variable** | **Non-serious manifestation of dengue**  **N=283** | **Serious manifestations of dengue**  **N=79** | **Total**  **N=362** | **OR [95CI]** | **p- value** |
| --- | --- | --- | --- | --- | --- |
| Age of patient, median years (IQR) | 13.0 (8.0 a 17.0) | 16.0 (9.0 to 27.0) | 13.0 (8.0 to 18.0) | 1.02 [1.01, 1.03] | 0.038 |
| Sex n(%) |  |  |  |  |  |
| Female | 125 (44.2) | 34 (43.0) | 159 (43.9) | - |  |
| Male | 158 (55.8) | 45 (57.0) | 203 (56.1) | 1.05 [0.63, 1.74] | 0.858 |
| Race n(%) |  |  |  |  |  |
| White | 54 (19.1) | 15 (19.0) | 69 (19.1) | - |  |
| Black | 20 (7.1) | 8 (10.1) | 28 (7.7) | 1.44 [0.51, 3.86] | 0.475 |
| Mix race | 209 (73.9) | 56 (70.9) | 265 (73.2) | 0.96 [0.52, 1.89] | 0.913 |
| Level of education n(%) |  |  |  |  |  |
| Less than Basic | 132 (46.6) | 33 (41.8) | 165 (45.6) | - |  |
| Basic/intermediate | 116 (41.0) | 31 (39.2) | 147 (40.6) | 1.07 [0.62, 1.85] | 0.812 |
| Advanced | 35 (12.4) | 15 (19.0) | 50 (13.8) | 1.72 [0.82, 3.47] | 0.140 |
| Place of origin n(%) |  |  |  |  |  |
| Urban | 274 (96.8) | 77 (97.5) | 351 (97.0) | - |  |
| Rural | 9 (3.2) | 2 (2.5) | 11 (3.0) | 0.79 [0.12, 3.15] | 0.767 |
| Occupation n(%) |  |  |  |  |  |
| House wive | 16 (5.7) | 6 (7.6) | 22 (6.1) | - |  |
| Employee | 32 (11.3) | 9 (11.4) | 41 (11.3) | 0.75 [0.23, 2.58] | 0.637 |
| Freelance work | 5 (1.8) | 3 (3.8) | 8 (2.2) | 1.60 [0.26, 8.87] | 0.590 |
| Unemployed/none | 38 (13.4) | 20 (25.3) | 58 (16.0) | 1.40 [0.49, 4.42] | 0.540 |
| Student | 192 (67.8) | 41 (51.9) | 233 (64.4) | 0.57 [0.22, 1.67] | 0.268 |
| Body mass index**^a^** n(%) |  |  |  |  |  |
| Underweight | 20 (7.1) | 4 (5.1) | 24 (6.6) | - |  |
| Normal | 114 (40.3) | 35 (44.3) | 149 (41.2) | 1.54 [0.54, 5.54] | 0.461 |
| Overweight | 61 (21.6) | 12 (15.2) | 73 (20.2) | 0.98 [0.30, 3.83] | 0.979 |
| Obese | 88 (31.1) | 28 (35.4) | 116 (32.0) | 1.59 [0.55, 5.81] | 0.431 |
| Any comorbidity n(%) | 41 (14.5) | 11 (13.9) | 52 (14.4) | 0.95 [0.45, 1.90] | 0.900 |
| Comorbidities n (%) |  |  |  |  |  |
| Pulmonary disease | 8 (2.8) | 0 (0.0) | 8 (2.2) |  |  |
| Cardiovascular disease | 5 (1.8) | 3 (3.8) | 8 (2.2) |  |  |
| Neurological disease | 10 (3.5) | 4 (5.1) | 14 (3.9) |  |  |
| Metabolic disease | 9 (3.2) | 1 (1.3) | 10 (2.8) |  |  |
| Kidney disease | 3 (1.1) | 1 (1.3) | 4 (1.1) |  |  |
| Immunosuppression | 5 (1.8) | 2 (2.5) | 7 (1.9) |  |  |
| Hematological disease | 1 (0.4) | 0 (0.0) | 1 (0.3) |  |  |
| None | 242 (85.5) | 68 (86.1) | 310 (85.6) |  |  |
| Time fever to consult (days), median (IQR) | 3.0 (1.0 to 4.0) | 2.0 (1.0 to 4.0) | 3.0 (1.0 to 4.0) | 0.96 [0.83, 1.10] | 0.569 |
| ***Clinical symptoms*** n(%) |  |  |  |  |  |
| Headache | 248 (87.6) | 72 (91.1) | 320 (88.4) | 1.45 [0.65, 3.69] | 0.392 |
| Retro-ocular pain | 183 (64.7) | 59 (74.7) | 242 (66.9) | 1.61 [0.93, 2.88] | 0.096 |
| Mialgias/ Arthralgias | 251 (88.7) | 72 (91.1) | 323 (89.2) | 1.31 [0.58, 3.35] | 0.536 |
| Rash | 151 (53.4) | 45 (57.0) | 196 (54.1) | 1.16 [0.70, 1.92] | 0.570 |
| Vomiting | 190 (67.1) | 54 (68.4) | 244 (67.4) | 1.06 [0.62, 1.83] | 0.838 |
| Abdominal pain | 215 (76.0) | 65 (82.3) | 280 (77.3) | 1.47 [0.79, 2.87] | 0.238 |
| Diarrhea | 111 (39.2) | 39 (49.4) | 150 (41.4) | 1.51 [0.91, 2.50] | 0.107 |
| Bleeding | 84 (29.7) | 37 (46.8) | 121 (33.4) | 2.09 [1.25, 3.48} | 0.005 |
| Petechiaes | 149 (52.7) | 42 (53.2) | 191 (52.8) | 1.02 [0.62, 1.69] | 0.935 |
| Edema | 50 (17.7) | 29 (36.7) | 79 (21.8) | 2.70 [1.55, 4.68] | <0.001 |
| ***Laboratory characteristics,*** *median (IQR)* |  |  |  |  |  |
| Leukocytes | 4.5 (2.9 to 6.4) | 4.6 (3.0 to 7.9) | 4.5 (3.0 to 7.0) | 1.05 [0.98, 1.11] | 0.130 |
| Neutrophils | 1.8 (1.1 to 3.3) | 1.6 (1.0 to 3.0) | 1.8 (1.0 to 3.2) | 1.02 [0.94, 1.11] | 0.554 |
| Lymphocytes | 1.6 (0.9 to 2.9) | 2.0 (1.1 to 3.1) | 1.7 (1.0 to 2.9) | 1.10 [0.96, 1.25] | 0.158 |
| Platelets | 113.0 (57.5 to 186.5) | 34.0 (23.0 to 84.0) | 90.5 (43.0 to 172.0) | 0.99 [0.99, 1.00] | <0.001 |
| ALT | 51.0 (26.0 to 115.7) | 74.0 (40.0 to 159.0) | 58.0 (27.5 to 121.5) | 1.002 [1.000, 1.003] | 0.030 |
| No. | 272 | 79 | 351 |  |  |
| AST | 91.5 (52.5 to 191.0) | 140.0 (97.5 to 294.0) | 107.0 (57.0 to 212.0) | 1.001 [1.000, 1.001] | 0.066 |
| No. | 272 | 79 | 351 |  |  |
| IgG n(%) |  |  |  |  |  |
| No. | 262 | 73 | 335 |  |  |
| Negative | 109 (41.6) | 14 (19.2) | 123 (36.7) | - |  |
| Positive | 153 (58.4) | 59 (80.8) | 212 (63.3) | 3.00 [1.64, 5.84] | <0.001 |
| Dengue serotype n(%) |  |  |  |  |  |
| Unknown | 123 (43.5) | 26 (32.9) | 149 (41.2) | 0.46 [0.26, 0.82] | 0.009 |
| DENV-1 | 26 (9.2) | 5 (6.3) | 31 (8.6) | 0.42 [0.13, 1.11] | 0.102 |
| DENV-2 | 79 (27.9) | 36 (45.6) | 115 (31.8) | - |  |
| DENV-3 | 55 (19.4) | 12 (15.2) | 67 (18.5) | 0.49 [0.22, 0.98] | 0.049 |
| DENV-4 | 0 (0.0) | 0 (0.0) | 0 (0.0) |  |  |
| Relevant dengue lineages n(%) |  |  |  |  |  |
| 1V_D.1 | 16 (5.7) | 3 (3.8) | 19 (5.2) | 0.86 [0.19, 2.78] | 0.818 |
| 2II_F.1.1.2 | 46 (16.3) | 21 (26.6) | 67 (18.5) | 2.09 [1.09, 3.98] | 0.025 |
| 2III_D.2 | 25 (8.8) | 12 (15.2) | 37 (10.2) | 2.20 [0.97, 4.79] | 0.051 |
| 3III_C.1 | 54 (19.1) | 12 (15.2) | 66 (18.2) | 1.02 [0.47, 2.08] | 0.962 |
| Other/unknown | 142 (50.2) | 31 (39.2) | 173 (47.8) | - | - |

**Sociodemographic and clinical characteristics of patients with serious and non-serious manifestations of dengue in cities other than Cali**

| **Variable** | **Non-serious manifestation of dengue**  **N=150** | **Serious manifestations of dengue**  **N=88** | **Total**  **N=238** | **OR [95CI]** | **p- value** |
| --- | --- | --- | --- | --- | --- |
| Age of patient, median years (IQR) | 12.0 (8.0 to 17.0) | 16.0 (10.0 to 29.5) | 13.0 (8.0 to 21.0) | 1.03 [1.01, 1.05] | 0.003 |
| Sex n(%) |  |  |  |  |  |
| Female | 68 (45.3) | 45 (51.1) | 113 (47.5) | - |  |
| Male | 82 (54.7) | 43 (48.9) | 125 (52.5) | 0.79 [0.47, 1.34] | 0.387 |
| Race n(%) |  |  |  |  |  |
| White | 12 (8.0) | 11 (12.5) | 23 (9.7) | - |  |
| Black | 13 (8.7) | 8 (9.1) | 21 (8.8) | 0.67 [0.20, 2.22] | 0.516 |
| Mix race | 125 (83.3) | 69 (78.4) | 194 (81.5) | 0.60 [0.25, 1.46] | 0.253 |
| Level of education n(%) |  |  |  |  |  |
| Less than Basic | 73 (48.7) | 35 (39.8) | 108 (45.4) | - |  |
| Basic/intermediate | 67 (44.7) | 41 (46.6) | 108 (45.4) | 1.28 [0.73, 2.24] | 0.393 |
| Advanced | 10 (6.7) | 12 (13.6) | 22 (9.2) | 2.50 [0.99, 6.48] | 0.053 |
| Place of origin n(%) |  |  |  |  |  |
| Urban | 122 (81.3) | 76 (86.4) | 198 (83.2) | - |  |
| Rural | 28 (18.7) | 12 (13.6) | 40 (16.8) | 0.69 [0.32, 1.40] | 0.318 |
| Occupation n(%) |  |  |  |  |  |
| House wive | 9 (6.0) | 12 (13.6) | 21 (8.8) | - |  |
| Employee | 13 (8.7) | 13 (14.8) | 26 (10.9) | 0.75 [0.23, 2.38] | 0.626 |
| Freelance work | 3 (2.0) | 4 (4.5) | 7 (2.9) | 1.00 [0.17, 6.16] | >0.999 |
| Unemployed/none | 22 (14.7) | 11 (12.5) | 33 (13.9) | 0.37 [0.12, 1.14] | 0.088 |
| Student | 103 (68.7) | 48 (54.5) | 151 (63.4) | 0.35 [0.13, 0.88] | 0.027 |
| Body mass index**^a^** n(%) |  |  |  |  |  |
| Underweight | 7 (4.7) | 9 (10.2) | 16 (6.7) | - |  |
| Normal | 66 (44.0) | 26 (29.5) | 92 (38.7) | 0.31 [0.10, 0.90] | 0.033 |
| Overweight | 30 (20.0) | 25 (28.4) | 55 (23.1) | 0.65 [0.20, 1.98] | 0.448 |
| Obese | 47 (31.3) | 28 (31.8) | 75 (31.5) | 0.46 [0.15, 1.38] | 0.168 |
| Any comorbidity n(%) | 19 (12.7) | 16 (18.2) | 35 (14.7) | 1.53 [0.73, 3.16] | 0.248 |
| Comorbidities n (%) |  |  |  |  |  |
| Pulmonary disease | 4 (2.7) | 4 (4.5) | 8 (3.4) |  |  |
| Cardiovascular disease | 3 (2.0) | 5 (5.7) | 8 (3.4) |  |  |
| Neurological disease | 5 (3.3) | 0 (0.0) | 5 (2.1) |  |  |
| Metabolic disease | 2 (1.3) | 3 (3.4) | 5 (2.1) |  |  |
| Kidney disease | 3 (2.0) | 2 (2.3) | 5 (2.1) |  |  |
| Immunosuppression | 2 (1.3) | 2 (2.3) | 4 (1.7) |  |  |
| Hematological disease | 0 (0.0) | 0 (0.0) | 0 (0.0) |  |  |
| None | 131 (87.3) | 72 (81.8) | 203 (85.3) |  |  |
| Time fever to consult (days), median (IQR) | 3.0 (1.0 to 4.0) | 2.0 (1.0 to 4.0) | 3.0 (1.0 to 4.0) | 0.99 [0.86, 1.14] | 0.937 |
| ***Clinical symptoms*** n(%) |  |  |  |  |  |
| Headache | 132 (88.0) | 78 (88.6) | 210 (88.2) | 1.06 [0.47, 2.50] | 0.883 |
| Retro-ocular pain | 108 (72.0) | 61 (69.3) | 169 (71.0) | 0.88 [0.49, 1.57] | 0.660 |
| Mialgias/ Arthralgias | 133 (88.7) | 84 (95.5) | 217 (91.2) | 2.68 [0.95, 9.57] | 0.085 |
| Rash | 85 (56.7) | 44 (50.0) | 129 (54.2) | 0.76 [0.45, 1.30] | 0.319 |
| Vomiting | 98 (65.3) | 65 (73.9) | 163 (68.5) | 1.50 [0.84, 2.72] | 0.173 |
| Abdominal pain | 124 (82.7) | 74 (84.1) | 198 (83.2) | 1.11 [0.55, 2.31] | 0.777 |
| Diarrhea | 58 (38.7) | 41 (46.6) | 99 (41.6) | 1.38 [0.81, 2.36] | 0.232 |
| Bleeding | 53 (35.3) | 36 (40.9) | 89 (37.4) | 1.27 [0.73, 2.17] | 0.391 |
| Petechiaes | 74 (49.3) | 52 (59.1) | 126 (52.9) | 1.48 [0.87, 2.54] | 0.146 |
| Edema | 29 (19.3) | 30 (34.1) | 59 (24.8) | 2.16 [1.19, 3.94] | 0.012 |
| ***Laboratory characteristics,*** *median (IQR)* |  |  |  |  |  |
| Leukocytes | 4.9 (3.1 to 6.9) | 5.6 (3.8 to 7.9) | 4.9 (3.3 to 7.1) | 1.06 [0.99, 1.13] | 0.073 |
| Neutrophils | 1.7 (1.1 to 2.7) | 2.0 (1.4 to 3.1) | 1.8 (1.2 to 2.9) | 1.09 [0.99, 1.19] | 0.057 |
| Lymphocytes | 2.2 (1.2 to 3.4) | 2.2 (1.4 to 3.4) | 2.2 (1.2 to 3.4) | 1.03 [0.89, 1.19] | 0.669 |
| Platelets | 65.5 (43.0 to 111.5) | 34.5 (23.0 to 55.7) | 52.0 (33.2 to 94.0) | 0.996 [0.992, 0.999] | 0.024 |
| ALT | 61.0 (34.0 to 130.0) | 56.5 (40.7 to 105.2) | 58.0 (35.5 to 118.5) | 0.999 [0.998, 1.001] | 0.438 |
| No. | 147 | 88 | 235 |  |  |
| AST | 115.0 (69.5 to 228.2) | 119.0 (69.5 to 187.5) | 116.0 (69.5 to 213.5) | 1.001 [0.999, 1.001] | 0.880 |
| No. | 148 | 88 | 235 |  |  |
| IgG n(%) |  |  |  |  |  |
| No. | 136 | 76 | 212 |  |  |
| Negative | 62 (45.6) | 29 (38.2) | 91 (42.9) | - |  |
| Positive | 74 (54.4) | 47 (61.8) | 121 (57.1) | 1.36 [0.77, 2.42] | 0.295 |
| Dengue serotype n(%) |  |  |  |  |  |
| Unknown | 71 (47.3) | 40 (45.5) | 111 (46.6) | 0.64 [0.35, 1.15] | 0.136 |
| DENV-1 | 16 (10.7) | 6 (6.8) | 22 (9.2) | 0.42 [0.14, 1.15] | 0.107 |
| DENV-2 | 42 (28.0) | 37 (42.0) | 79 (33.2) | - |  |
| DENV-3 | 19 (12.7) | 5 (5.7) | 24 (10.1) | 0.30 [0.09, 0.82] | 0.028 |
| DENV-4 | 2 (1.3) | 0 (0.0) | 2 (0.8) |  |  |
| Relevant dengue lineages n(%) |  |  |  |  |  |
| 1V_D.1 | 10 (6.7) | 4 (4.5) | 14 (5.9) | 0.79 [0.21, 2.51] | 0.705 |
| 2II_F.1.1.2 | 25 (16.7) | 20 (22.7) | 45 (18.9) | 1.58 [0.79, 3.16] | 0.193 |
| 2III_D.2 | 11 (7.3) | 16 (18.2) | 27 (11.3) | 2.88 [1.24, 6.88] | 0.015 |
| 3III_C.1 | 17 (11.3) | 4 (4.5) | 21 (8.8) | 0.46 [0.13, 1.35] | 0.191 |
| Other/unknown | 87 (58.0) | 44 (50.0) | 131 (55.0) | - |  |

10. Lines 393-402. Cites several studies, but references only one. Further discuss the relationship between education and disease severity based on scientific findings.

**Response:** Considering the lack of association in the final model between level of education and serious manifestations, the “hygiene hypothesis paragraph” has been deleted from the discussion.

11. Discuss why DENV2 was not associated with dengue severity in the analysis performed according to WHO stratification.

**Response:** Thank you for pointing this out. Using the WHO 2009 clinical classification, we identified 22 severe dengue cases and 578 non-severe cases. Only 10 of the 22 severe cases had an identified serotype, 7 of which were DENV-2. Given the small number of severe cases and limited serotyping, estimates of serotype–severity associations are imprecise (as reflected by wider confidence intervals), so these results should be interpreted cautiously; we note this in the Results section. Because evaluation of severe dengue according to the 2009 WHO classification was not a prespecified objective of this study, and low case numbers, we did not expand these analyses in the manuscript, but we are happy to provide additional detail if the editor or reviewers feel it would be helpful.

**Conclusions**

Reviewer #1

1. The discussion should acknowledge the existence of studies exploring the same research question in other countries.

**Response:** Agree. In the Discussion, we acknowledge that other studies — including a systematic review covering multiple Latin American countries — have evaluated risk factors for severe dengue using the 2009 WHO classification (lines 327-328). In various paragraphs we also cite studies assessing risk factors for severe dengue under WHO classification. However, our study is distinct in that it evaluates risk factors using a newly proposed dengue framework,^1,2^ and to our knowledge, no prior research has examined risk factors under this novel classification.

^1^ Gómez-Zambrano M, Torres-Hernández D, Murillo-Ortiz MA, Hurtado IC, Dávalos DM, Cantor E, et al. Different clinical severity and outcomes in a cohort of patients with dengue with warning signs in an endemic Latin American city. Open Forum Infectious Diseases. 2025.

^2^ Tomashek KM, Wills B, See Lum LC, Thomas L, Durbin A, Leo YS, et al. Development of standard clinical endpoints for use in dengue interventional trials. PLoS Negl Trop Dis. 2018;12(10):e0006497.

2. Furthermore, any discussion regarding the different effects of serotype in the current study should recognize that nearly half of the cases lacked a defined serotype.

**Response:** Thank you, we agree. We have highlighted the lack of a defined serotype in different parts of the discussion, including in the limitations section.

Reviewer #2

1. The authors’ statement that "a vaccine is needed that is safe and effective regardless of prior dengue infection" seems overly ambitious. In the study, participants identified as having "prior dengue infection (IgG positive)"—but how can one distinguish whether the IgG positivity resulted from prior natural infection or from previous vaccination.

**Response:** We appreciate this observation. Considering all the reviewers’ suggestions, the lack of an effect of previous dengue exposure is now evidenced in the multivariate model and in the interaction model.

We now broadened our conclusion to include not only vaccines, but also other tetravalent preventive measures (lines 411-414). Considering that no dengue vaccines were available during the study period, as now mentioned in methods (lines 106-109) dengue IgG most likely resulted from previous exposure. Nevertheless, we acknowledge the limited specificity of the VIDAS IgG test to document prior dengue exposure in the limitations section.

**Summary and General Comments**

Reviewer #1: Minors

1. Abstract. Report Confidence intervals together with OR, not p-values

**Response:** In the original version, p-values were reported for the results of the interaction model. This has now been removed and replaced by the sentence “History of dengue infection showed no significant effect on the risk of serious clinical manifestations across the different serotypes”. The abstract now reflects the main findings from the new regression model, as suggested by all reviewers (lines 50-51).

2. Introduction. The first paragraph is vague. “Models indicate that case numbers will continue to increase in the coming years unless effective measures are widely implemented” Which type of model? Increase everywhere or only in Latin America? etc, provide more information

**Response:** We thank the reviewer for this helpful suggestion. Different statistical models, including boosted regression tree models or a generalized additive mixed model with a negative binomial distribution, have been employed to estimate future dengue circulation in different regions of the world. We have tried to be more specific in the first paragraph of the introduction. It now reads “models using different statistical techniques estimate that case numbers will continue to increase in various world regions in the coming years unless effective measures are widely implemented”, and we can provide further details of the models if the editor or reviewer prefer. Two new references were included.

3. There are some studies evaluating dengue with/without warning signs. Example:
   “Gonçalves, Bianca De Santis, et al. "Factors predicting the severity of dengue in patients with warning signs in Rio de Janeiro, Brazil (1986–2012)." Transactions of The Royal Society of Tropical Medicine and Hygiene 113.11 (2019): 670-677.”

Pinto, Rosemary Costa, et al. "Mortality predictors in patients with severe dengue in the State of Amazonas, Brazil." PloS one 11.8 (2016): e0161884.
It should be rephrased and take into account previous studies in the introduction (third paragraph)

**Response:** We thank the reviewer for this valuable suggestion. We have revised the third paragraph of the introduction to acknowledge the work of Gonçalves et al. who examined risk factors for dengue with warning signs (Pinto et al examined factors associated with death). We agree that their contributions are important in contextualizing our research question. However, as described in the text, our study differs from these prior investigations by focusing specifically on a subgroup of patients within the dengue with warning signs category which we designate as “serious manifestations of dengue”. This represents a novel classification not previously distinguished in earlier studies and is defined as severe dengue, or DwWS that progressed to a more severe disease course (as described in references 6 and 11 in the current version of the manuscript). According to our knowledge and literature review, this is the first study evaluating the risk factors for serious manifestations. Thus, our study extends beyond the existing body of work by addressing risk factors in this particularly critical and underexplored patient population. A detailed analysis of the importance and limitations of this category is reviewed in reference 11.

We have modified the third paragraph in the introduction, hoping to convey a clearer message.

Reviewer #2

1. This study is based on data collected during the large-scale dengue outbreak at HUV Hospital in Colombia (2023–2024), aiming to identify risk factors associated with "serious manifestations" of dengue. I believe the article has several issues in terms of methodological design, statistical modeling, and interpretation of results.

**Response:** We thank all the reviewers for their comments and suggestions that have helped us improved the quality of the data and clarity of the message.

Reviewer #3

1. The causality of DENV2 with disease severity should be further discussed and explored if this is the focus of the study.

**Response:** We agree with the reviewer. However, the study was not designed to estimate causal effects. Following this reviewer’s previous suggestion, dengue serotypes were not recoded and now serotype 2 is no longer compared against “others”. In addition, the interaction model was modified: We assessed the interaction between all serotypes and history of dengue infection (IgG) with respect to dengue severity, incorporating in the analysis age, sex, city of origin, education level, and occupation. In summary, the focus of the study was not to explore DENV-2 as the cause of severe disease and we hope to have made this more clear in the current version.
