# Supplementary material for: Epidemiology and severity risk factors of dengue virus infection during the 2023-2024 outbreak in Colombia
Source: PLoS Negl Trop Dis. 2025 Dec 5;19(12):e0013115. doi: 10.1371/journal.pntd.0013115 (PMC12694831; doi:10.1371/journal.pntd.0013115)
Supplement: Attachment — Submitted filename: Response to reviewer_Nov2025.docx [file pntd.0013115.s004.docx]

**Reviewer #1**

- **Minor comments on the Table 1/2 lack the measurement unit of the laboratory characteristics.**

**RESPONSE:**

Thank you for highlighting the lack of measurement units. This has now been corrected.

- **Also, the regression with laboratory characteristics as a continuous variable is not informative, as there is a range of values normal for each characteristic. A better approach would be to characterise as below/normal/above the reference values for each characteristic**

**RESPONSE:**

Thank you for this comment. From the study planning, we discussed wether to analyse laboratory characteristics as continuous or categorical variables.

We decided to follow the general statistical advice -- to keep continuous exposure variables as continuous in statistical analyses (1,2,3). According to the literature, converting a continuous variable into discrete categories can lead to loss of information and statistical power as the granularity of the original data is reduced. This is often equivalent to a significant reduction in effective sample size (4,5). This loss impairs the ability to detect true associations and can lead to less precise estimates.

A further important disadvantage of categorization is that it does not make use of within-category information: Everyone above or below the reference cut-point is treated as equal, yet their prognosis may vary considerably. It also assumes abrupt changes at boundaries, which rarely reflects biological reality and can distort the true relationship between the variable and outcome. These are some of the reasons why different medical disciplines, including the CHEST guidelines for statistical analysis and reporting recommend to avoid Categorization of Continuous Variables (6,7).

Finally, the laboratory parameters used in this study, and the different laboratory cutpoints that have been used in previous studies are different (8-10), so all of these results can not easily be compared, hampering meta-analysis of observational studies.

For example, in a regression analysis to develop a prognostic model for patients with primary biliary cirrhosis, a carefully developed model with bilirubin as a continuous explanatory variable explained 31% more of the variability in the data than when bilirubin distribution was split at the median (11).

However, if the editor or reviewer prefer, we would proceed with re-coding the continuous variables into two (liver function tests, platelets) or three (white blood cells) categories.

References

1. Alt Altman DG, Royston P. The cost of dichotomising continuous variables. BMJ. 2006 May 6;332(7549):1080. doi: 10.1136/bmj.332.7549.1080.

2. van Walraven C, Hart RG. Leave 'em alone - why continuous variables should be analyzed as such. Neuroepidemiology. 2008;30(3):138-9. doi: 10.1159/000126908.

3. Collins GS, Ogundimu EO, Cook JA, Manach YL, Altman DG. Quantifying the impact of different approaches for handling continuous predictors on the performance of a prognostic model. Stat Med. 2016 Oct 15;35(23):4124-35. doi: 10.1002/sim.6986.

4. Polley MC, Dignam JJ. Statistical Considerations in the Evaluation of Continuous Biomarkers. J Nucl Med. 2021 May 10;62(5):605-611. doi: 10.2967/jnumed.120.251520.

5. Naggara O, Raymond J, Guilbert F, Roy D, Weill A, Altman DG. Analysis by categorizing or dichotomizing continuous variables is inadvisable: an example from the natural history of unruptured aneurysms. AJNR Am J Neuroradiol. 2011 Mar;32(3):437-40. doi: 10.3174/ajnr.A2425.

6. Kattan MW, Vickers AJ. Statistical Analysis and Reporting Guidelines for CHEST. Chest. 2020 Jul;158(1S):S3-S11. doi: 10.1016/j.chest.2019.10.064.

7. Salis Z, Gallego B, Sainsbury A. Researchers in rheumatology should avoid categorization of continuous predictor variables. BMC Med Res Methodol. 2023 Apr 26;23(1):104. doi: 10.1186/s12874-023-01926-4.

8. Lee IK, Liu JW, Chen YH, Chen YC, Tsai CY, Huang SY, Lin CY, Huang CH. Development of a Simple Clinical Risk Score for Early Prediction of Severe Dengue in Adult Patients. PLoS One. 2016 May 3;11(5):e0154772. doi: 10.1371/journal.pone.0154772.

9. Islam S, Khan MAS, Badal MFA, Khan MZI, Gozal D, Hasan MJ. Clinical and hematological profiles of children with dengue residing in a non-endemic zone of Bangladesh. PLoS Negl Trop Dis. 2022 Oct 10;16(10):e0010847. doi: 10.1371/journal.pntd.0010847.

10. Jain S, Mittal A, Sharma SK, Upadhyay AD, Pandey RM, Sinha S, Soneja M, Biswas A, Jadon RS, Kakade MB, Dayaraj C. Predictors of Dengue-Related Mortality and Disease Severity in a Tertiary Care Center in North India. Open Forum Infect Dis. 2017 May 5;4(2):ofx056. doi: 10.1093/ofid/ofx056.

11. Royston P, Altman DG, Sauerbrei W. Dichotomizing continuous predictors in multiple regression: a bad idea. Stat Med. 2006 Jan 15;25(1):127-41. doi: 10.1002/sim.2331.
